# Why Do Chinese Employees Complain at the Workplace? An Exploratory Study Based on the Theory of Work Adjustment

**DOI:** 10.3389/fpsyg.2022.920041

**Published:** 2022-07-15

**Authors:** Shaofeng Yuan, Ying Gao

**Affiliations:** ^1^Business School, Liaoning University, Shenyang, China; ^2^Sun Wah International Business School, Liaoning University, Shenyang, China

**Keywords:** causes of employee complaints, measurement scale, Chinese employee, person-environment fit, interpersonal relationships

## Abstract

This study aims to investigate the causes of workplace complaints among Chinese employees and to develop a scale to measure them, drawing on the theory of work adjustment. We first obtained 49 items regarding employees' complaints following rigorous item generation and refinement procedures. Then, we conducted a survey with convenience sampling and obtained a sample of 268 employees. The exploratory factor analysis based on this sample generated a six-factor solution that explained 65.85% of the variance. The six factors include four person-environment (P-E) interactional factors, namely, dissatisfaction due to (a) interpersonal relationships; (b) management systems; (c) work conditions; and (d) authoritarian leadership; and two P-E misfit factors, namely, perceived misfit regarding (e) work content; and (f) job responsibilities. Furthermore, we obtained another sample of 349 employees through snowball sampling, on which we further validated the six-correlated-factor solution through confirmatory factor analysis. This study contributes to the literature by identifying causes of Chinese employees' complaints different from those attributed to their counterparts in Western cultures. This outcome particularly reveals that “dissatisfaction with interpersonal relationships” with colleagues was the leading cause of complaints among Chinese employees, rather than the “misfit between employees' needs and organizational rewards” revealed by Western culture-based studies. Both our findings and the scale we developed have practical implications for companies that employ Chinese employees.

## Introduction

Chinese employees' complaints in the workplace are a universal phenomenon. An investigation based on a sample of over 5,000 Chinese employees, which was conducted by a leading recruitment website in China, indicated that 65.7% of workers in the sample usually complained 1–5 times, 13.8% complained 6–10 times, 3.7% complained 11–15 times, and 4.8% complained over 15 times a day in their workplace, and 80.5% of their complaints were targeted at their jobs (cf. Lu and Liu, 2016). Employee complaints are expressions of work- and organization-related dissatisfaction (Kowalski, 1996; Walker and Hamilton, 2011a). Individually, employee complaints are usually accompanied by negative outcomes such as absenteeism, turnover, lowered productivity, and disruptive workplace behaviors (Bamberger et al., 2008; Walker and Hamilton, 2011a). Collectively, employee complaints might result in negative outcomes such as poor firm performance (Dimmock and Gerken, 2018), overall lowered organizational commitments (Gutierrez et al., 2012), and decreased intention of voice (Wu and Ma, 2022). Thus, identifying the causes of Chinese employees' complaints and developing a scale to measure them are important issues for firms.

A vast majority of prior studies addressing the causes of employee complaints have been conducted in Western cultures. They have documented that employees' perceived misfit with work content, and/or job responsibilities could result in employees' grievances or complaints (Bacharach and Bamberger, 2004; Furåker, 2009; Branham, 2012; Warr and Inceoglu, 2012). Moreover, employees' dissatisfaction with supervision, work conditions (Bacharach and Bamberger, 2004), managerial monitoring (Kleiner et al., 1995), unfair treatment (Boswell and Olson-Buchanan, 2004), and employment rights disputes (Walker and Hamilton, 2011b) have also been affirmed as causes of complaints. While all of these factors can lead to employee complaints, more studies (e.g., Piasentin and Chapman, 2006; Westover and Taylor, 2010; Rubin and Edwards, 2020) based on the theory of work adjustment suggested that the misfit between employees' need and organizations' reward was the principal cause of Western employees' complaints in the workplace.

Given that there are significant differences between the West and China regarding employees' personalities, cultural values, economic systems, laws, and industrial relations (Dessler and Tan, 2008; Xiao and Cooke, 2012), the causes of Chinese employees' complaints might significantly differ from those of their counterparts in Western cultures. However, few prior studies have specifically investigated the causes of Chinese employees' complaints in the workplace (Lu and Liu, 2016).

To fill this gap, this study aims to investigate the major causes of Chinese employees' complaints and develop a scale to measure them. The theory of work adjustment (Dawis and Lofquist, 1984)—a theory concerning a person (P) in a work environment (E) and the fit and interactions between the P and the E—was widely used in organizational psychology to explain employees' job-related behaviors (Bayl-Smith and Griffin, 2015; Guan et al., 2021), this study follows this theory to explore the causes of employees' complaints. Through two survey-based studies, this research identifies six crucial causes of Chinese employees' complaints, four of which are P-E interactional factors: dissatisfaction with (a) interpersonal relationships; (b) management systems; (c) work conditions; and (d) authoritarian leadership; the remaining two are P-E misfit factors: perceived misfit regarding (e) work content; and (f) job responsibilities. The findings show that the first factor (i.e., dissatisfaction with interpersonal relationships) contributed the most variance in complaints.

This study makes the following contributions. First, it reveals six crucial causes of Chinese employees' complaints. In particular, it shows that the P-E interactional factor, i.e., dissatisfaction with interpersonal relationships with colleagues, was the leading factor (accounting for 34.25% of the variance) of Chinese employees' complaints. In contrast, prior Western culture-based studies (cf. Piasentin and Chapman, 2006; Westover and Taylor, 2010; Rubin and Edwards, 2020) affirmed that the misfit between employees' needs and organizations' rewards was a key factor leading to employees' complaints. Moreover, this study develops a scale for employers to measure those causes and thus can help companies address their Chinese employees' complaints.

The rest of this study is divided as follows. The next section provides the theoretical background and research questions. Then, in Study 1, item generation and initial reduction procedures are presented, and an exploratory factor analysis based on a sample of 268 employees was conducted to further remove unrepresentative items. In subsequent Study 2, confirmatory factor analysis based on another sample of 349 employees was conducted to validate the scale. Finally, the article ends with a general discussion.

## Theoretical Background and Research Questions

### The Theory of Work Adjustment

Extensive prior studies in the field of organizational psychology applied the theory of work adjustment to explain employees' satisfied or dissatisfied behaviors in the workplace (Bayl-Smith and Griffin, 2015; Guan et al., 2021). It can also provide explanations for the possible causes of Chinese employees' complaints in their workplace. This theory is one kind of P-E theory (Dawis and Lofquist, 1984; Guan et al., 2021). In the workplace, P refers to the employee, and E refers to the work environment of an organization (e.g., job requirements, work conditions, interpersonal relationships, performance appraisal systems, payment systems, incentive systems). The basic proposition of this theory is that although the P and E variables can contribute to the explanation of employees' behavior or behavioral outcomes, it is a particular P-E combination that will best explain the specific behavior or behavioral outcome (Dawis, 2005).

Two constructs, fit and interaction, were used to denote the P-E combination. Fit is defined as the degree to which the P and E characteristics match (Kristof-Brown et al., 2005). The P characteristics include an employee's physiological or psychological needs, values, goals, abilities, or personalities, while the E characteristics include intrinsic and extrinsic rewards, job demands, cultural values, or characteristics of other individuals and collectives in the P's work environment (Kristof-Brown et al., 2005). By matching the right employee with the right job, employees can achieve a balanced state that leads to maintenance behavior (e.g., commitment to the organization). Otherwise, a misfit between an employee and a job position will result in his or her behaviors changing the situation (adjustment behavior).

Interaction refers to a P's and an E's actions and reactions in relation to one another in a mutual give and take (Dawis, 2005). As living organisms, employees (P) have needs (e.g., physiological and psychological needs) that must be met, most of them through the work environment (E). Employees have abilities (e.g., work skills) that enable them to meet these needs, and most employee behaviors in interacting with the work environment involve meeting these needs. Moreover, the work environment (E, in parallel with P) has demands (e.g., job requirements, firm norms, and role expectations) that must be met and supplies (e.g., payment, prestige, and working conditions) that enable it to meet its demands.

In the process of fulfilling mutual requirements, satisfaction or dissatisfaction occurs for employees and organizations. Satisfaction for both employees and organizations will lead to behaviors that maintain mutual interaction (maintenance behavior), but dissatisfaction will result in behaviors that change the situation (adjustment behavior).

Accordingly, a misfit between an employee and an organization and the absence of mutual satisfaction in P-E interactions will lead to adjustment behaviors to change the situation (Guan et al., 2021). One adjustment behavior of dissatisfied employees is to complain to management (Dawis, 2005) or others in their work environment. Thus, factors that lead to the following two situations could cause employees to complain: (a) there is a misfit between the employee and the organization, for instance, an employee's abilities are greater or less than the job requirements; and (b) there is dissatisfaction in the P-E interactions; for example, an employee's physiological or psychological needs are not met in the organization.

### Brief Review of the Causes of Employees' Complaints

We adopted the P-E combination perspective to review prior studies. Concerning the P-E misfit factors, some prior studies (e.g., Bacharach and Bamberger, 2004; Furåker, 2009; Branham, 2012; Warr and Inceoglu, 2012) from Western cultures have indicated that employees' perceived misfit with work content and job responsibilities could result in their dissatisfaction and subsequent behaviors (e.g., complaints). However, more studies (e.g., Piasentin and Chapman, 2006; Westover and Taylor, 2010; Rubin and Edwards, 2020) based on the theory of work adjustment supported that the misfit or incongruence between employees' needs and organizations' rewards was the principal factor that determined employees' dissatisfaction and subsequent workplace complaints.

Regarding the P-E interactional factors, prior studies have documented that dissatisfaction with supervision, work conditions (Bacharach and Bamberger, 2004), managerial monitoring (Kleiner et al., 1995), unfair treatment (Boswell and Olson-Buchanan, 2004), and employment rights disputes (Walker and Hamilton, 2011b) could result in employee dissatisfaction and complaints. Additionally, some theoretical articles (e.g., Barrick et al., 2013; Carnevale and Hatak, 2020) also showed that employees' perceived incongruence in work relationships with others could result in employee dissatisfaction and thus might further trigger their complaints.

Drawing on the theory of work adjustment theory and prior studies, this study specifically sheds light on the following questions:

**RQ1**: What specific misfit factors between P and E resulted in Chinese employees' complaints in the workplace?
**RQ2**: What specific dissatisfactory factors in P-E interactions resulted in Chinese employees' complaints?
**RQ3**: Are there any significant differences between China and the West in terms of their employees' complaints causes?

**Figure 1:**
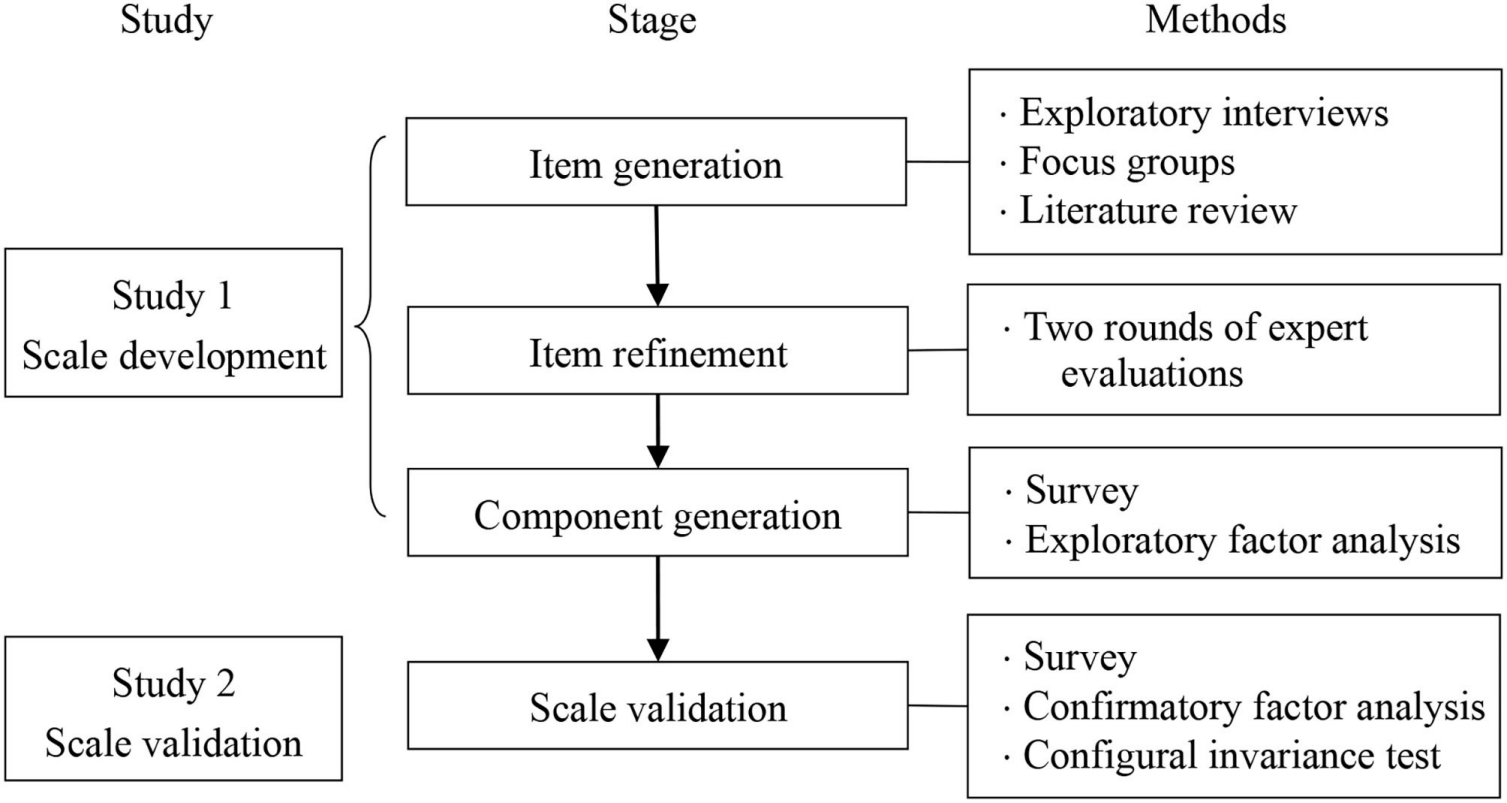
The research procedures.

Two studies were conducted to explore the causes of workplace complaints among Chinese employees and to answer the three research questions above. Study 1 aimed to develop a scale that could describe the main components of the causes of employees' complaints and assess the internal consistency reliability of the components of this scale. Study 2 aimed to test the validity of this scale with a sample from another survey. The research procedures are depicted in Figure 1. We designed these procedures based on the prior scaling literature (Netemeyer et al., 2003) and scale development research (e.g., Zhang et al., 2011, 2021). IBM SPSS software, versions 25.0, and LISREL software, versions 8.7, were used to analyze the data in Studies 1 and 2, respectively.

## Study 1: Scale Development

This study specifically included three stages. The first was the “item generation” stage, in which we generated a pool of 75 items on the causes of employee complaints through exploratory interviews, focus groups, and a literature review. The second was the “item refinement” stage, in which we conducted two rounds of expert evaluations to assess the content validity of each item. After this stage, we obtained 49 items with initial content validity. The third was the “component generation” stage. In this stage, we conducted a survey and obtained 268 valid responses, and then we conducted exploratory factor analysis to generate components of the causes of employee complaints.

### Item Generation

First, an original pool of 75 items regarding the causes of employee complaints was generated from exploratory interviews, focus groups, and a review of related prior studies. The exploratory interviews were conducted with 24 employees, 21 of whom worked in the IT, auto parts, electric power, management consulting, mobile communication, steel smelting, and real estate industries, respectively; the remaining three worked in the retail (2) and higher education (1) industries. The focus groups were conducted among 20 MBA students (employees of different companies) at a comprehensive university in northeast China.

### Item Refinement

Two associate professors and two Ph.D. students majoring in human resource management were invited to evaluate the understandability, clarity, and similarity of the 75 items generated from the previous stage. Before the evaluation procedure, they were told about the research's purposes and given the definition of the causes of employees' complaints. A revised pool of 62 items was obtained after eliminating ambiguous and essentially identical items based on the four judges' evaluations.

Next, three additional evaluators (Ph.D. students) were invited to evaluate the representativeness of each item according to the procedure recommended by Hinkin (1998). They were also told of the research purposes, given the definition of the causes of employees' complaints, and showed examples of these causes. They then evaluated each item on the following scale: 1 = *clearly representative*, 2 = *partially representative*, 3 = *not representative at all*. Only items judged as clearly represented by at least two evaluators were retained. The judgment results indicated that 38 items were “clearly representative” by all three evaluators, and 11 items were “clearly representative” by two of the three evaluators. Thirteen items were eliminated in this process, leaving 49 items for the following analysis.

### Component Generation

#### Subjects

Given the exploratory nature of this study and with reference to prior scale development studies (e.g., Zhang et al., 2011, 2021), a convenience sample was collected at this stage. Specifically, both online and offline data collection was used in this study. For the online part of the study, after designing the questionnaire on the Quatrics platform, links were delivered to employees in various industries. Of the 193 individuals who began the online survey, 157 finished it. For the offline part of the study, 125 printed questionnaires were distributed to MBA students of a leading university in northeastern China; 65 of them were distributed in marketing classes, and the other 60 were distributed in strategic management classes. In total, 111 valid responses were returned. All the participants provided informed consent. Of the 268 individuals who participated (including both offline and online participants), 47.0% were men, 50.0% were women, and 3.0% did not report their gender; 11.6% had a junior college degree, 34.0% had a bachelor's degree, 51.8% had a master's degree or above, and 2.6% did not report their education level; 30.7% worked in manufacturing, 26.8% in IT, 30.2% in services, 6.1% in energy, 3.2% in education, and 3.0% did not report their industries; 16.8% worked in a junior position, 52.6% worked in an intermediate position, and 30.6% worked in a senior management or technical position. The participants' ages ranged from 22 to 57 (*M*_*age*_ = 29.63, *SD* = 5.00).

#### Measures

The measurement included 49 items obtained from the second stage (i.e., item refinement) of this study. Participants answered each item on a 7-point scale, with 1=*completely disagree* and 7= *completely agree*.

### Results

Before the formal analysis, possible differences between the online (*N* = 157) and offline (*N* = 111) datasets regarding participants' responses on the 49 items were examined. The results of a one-way analysis of variance (ANOVA) showed that participants' responses on most of the items (44 out of 49) were not significantly different (*p* > 0.05) between the online and offline datasets; we combined these two datasets in the following analyses.

Next, the 49 items were factor-analyzed through principal component analysis with varimax rotation. The results showed that the Kaiser–Meyer–Olkin value reached 0.90, and Bartlett's test of sphericity showed statistical significance (*p* < 0.001), indicating that the data were suitable for factor analysis. Nine components with eigenvalues over 1 were obtained, and they explained 69.22% of the cumulative variance.

Of these items, those with factor loadings >0.4 on two or more intended components and those that did not load on any components at the criterion of 0.4 were eliminated (cf. Zhang et al., 2011). Only items with high factor loadings on one component but low loadings on other components were selected after this elimination process. A second-factor analysis that included the retained items was conducted, and the results showed a six-factor solution that accounted for 65.85% of the total variance. Factors 1 to 6 contributed 34.25, 8.90, 7.91, 6.06, 4.62, and 4.12% of the variance, respectively. The final six-factor solution with factor loadings, along with the commonalities, the eigenvalues, and the total variance explained, is shown in Table 1.

**Table 1:** Measurement of Chinese employee complaint causes.

**Factors and Items**	**Loadings**						**Co**
	**1**	**2**	**3**	**4**	**5**	**6**	
**Factor 1: Dissatisfaction with interpersonal relationship**							
Sometimes, some of my colleagues plot against me in my work.	**0.78**	0.07	0.12	0.22	0.04	0.12	0.69
During my work, useful information is deliberately withheld sometimes.	**0.77**	0.20	0.06/	−0. 04	0.18	0.21	0.72
At work, I am often misunderstood by my colleagues.	**0.73**	0.06	0.28	0.11	0.11	0.14	0.66
In my company, some colleagues harm others without benefiting themselves.	**0.71**	0.11	0.17	0.16	0.04	0.11	0.59
At work, I always feel isolated or neglected.	**0.67**	0.20	0.14	−0.08	0.19	0.28	0.62
In my company, intriguing against each other among employees is what the supervisors want.	**0.67**	0.20	0.04	0.07	0.06	0.25	0.56
I always have to brag to others about my contributions at work.	**0.62**	0.23	0.29	0.07	0.01	0.07	0.54
**Factor 2: Perceived misfit with work content**							
My work content is relatively simple and lacks challenges.	0.20	**0.77**	−0. 03	−0. 19	0.17	0.05	0.71
I find it difficult to achieve my career goals at current company.	0.14	**0.73**	0.28	0.16	0.06	0.04	0.65
My work content is always the same, I cannot learn anything new.	0.21	**0.71**	0.03	0.07	0.19	0.18	0.62
I am not interested in my work.	0.28	/**0.68**	0.07	0.02	0.09	0.02	0.56
The training in my company is inadequate.	−0. 03	**0.65**	0.31	0.15	0.08	0.24	0.61
**Factor 3: Dissatisfaction with management system**							
The job description of my position is ambiguous.	0.25	0.22	**0.76**	0.03	0.21	0.08	0.74
Objectives and responsibilities are ambiguous in my company.	0.19	0.15	**0.72**	0.15	0.34	0.11	0.73
I'm working under multiple managers.	0.38	0.06	**0.63**	0.14	0.05	0.25	0.63
The process of my work is not standardized.	0.18	0.15	**0.55**	0.24	0.11	0.27	0.51
**Factor 4: Perceived misfit with job responsibilities**							
My workload is heavy.	0.06	0.00	0.16	0**.80**	−0. 04	0.07	0.68
My job is of great responsibility.	0.06	−0. 14	−0. 18	/**0.79**	0.12	0.02	0.70
I always feel pressed for time completing tasks because they are assigned late.	0.13	0.12	0.31	**0.70**	0.10/	0.14	0.65
In my work, the inputs outweigh the outcomes.	0.21	0.27	0.22	**0.65**	0.08	0.05	0.59
**Factor 5: Dissatisfaction with work conditions**							
The surroundings of my company are poor.	0.15	0.17	0.12	0.02	**0.85**	0.15	0.81
The traffic is inconvenient at my workplace.	0.06	0.03	0.16	0.11	**0.82**	0.01	0.72
The decoration in my office is in poor condition.	0.15	0.28	0.20	0.08	**0.68**	0.16	0.64
**Factor 6: Dissatisfaction with authoritarian leadership**							
In my company, channels for employees to feedback problems to managers are lacking.	0.28	0.12	0.22	0.08	0.13	**0.78**	0.77
In my company, communications are lacking between supervisors and employees.	0.39	0.14	0.08	0.05	0.11	**0.75**	0.75
In my company, the supervisors are dictatorial and undemocratic.	0.32	0.17	0.26	0.19	0.10	**0.69**	0.72
Eigenvalue	8.91	2.31	2.06	1.58	1.20	1.07	
Contribution rate (%)	34.25	8.90	7.91	6.06	4.62	4.12	

*Co, Communalities; Extract, principal component analysis; Rotation, Varimax*.

The results in Table 1 show that the first factor consists of 7 items^1^ that describe employees' dissatisfaction with interpersonal relationships with colleagues (e.g., “Sometimes, some of my colleagues plot against me in my work”). We named it “dissatisfaction with interpersonal relationships”. The second factor, which includes 5 items, describes employees' perceived misfit with work content (e.g., “My work content is relatively simple and lacks challenges”), we named it “perceived misfit with work content”.

The third and fourth factors consist of 4 statements that reflect employees' discontentment with low-quality management (e.g., “The job description of my position is ambiguous”) and work overload (e.g., “My workload is heavy”) in their companies, representing the “dissatisfaction with management system” and “perceived misfit with job responsibilities” dimensions, respectively. The fifth and sixth factors consist of 3 statements that reflect employees' discontentment with poor work environments (e.g., “The surroundings of my company are poor”) and authoritarian leadership (e.g., “In my company, channels for employees to feedback problems to managers are lacking”) in their companies, representing the “dissatisfaction with work conditions” and “dissatisfaction with authoritarian leadership” dimensions, respectively.

Furthermore, the internal consistency of the scale was examined. The Cronbach's alpha of the six factors is 0.89, 0.82, 0.81, 0.77, 0.80, and 0.84 (see Table 2). The corrected item-total correlations of 26 items were no less than 0.5. These results indicate that the scale has good internal consistency.

**Table 2:** Mean, standard deviation (SD), Cronbach's alpha, and Pearson's correlations of the scale components.

**Scale dimensions**	**Mean**	**SD**	**1**	**2**	**3**	**4**	**5**	**6**
1. Dissatisfaction with interpersonal relationship	3.05	1.19	**0.89**					
2. Perceived misfit with work content	3.75	1.29	0.46^**^	**0.82**				
3. Dissatisfaction with management system	3.43	1.36	0.58^**^	0.46^**^	**0.81**			
4. Perceived misfit with job responsibilities	4.21	1.27	0.31^**^	0.20^**^	0.40^**^	**0.77**		
5. Dissatisfaction with work conditions	3.19	1.49	0.34^**^	0.38^**^	0.46^**^	0.23^**^	**0.80**	
6. Dissatisfaction with authoritarian leadership	3.44	1.46	0.63^**^	0.42^**^	0.56^**^	0.31^**^	0.36^**^	**0.84**

** *p < 0.01; The bold values on the diagonal are the Cronbach's alpha values*.

The results of the correlation analysis based on the means of each factor's items (see Table 2) reveal that the Pearson coefficients between “dissatisfaction with interpersonal relationships” and “dissatisfaction with authoritarian leadership” (*r* = 0.63, *p* < 0.01), between “dissatisfaction with interpersonal relationships” and “dissatisfaction with management system” (*r* = 0.58, *p* < 0.01), and between “dissatisfaction with authoritarian leadership” and “dissatisfaction with management system” (*r* = 0.56, *p* < 0.01) were relatively high, and the other correlation coefficients were moderately high (*r* ranging from 0.20 to 0.46).

## Study 2: Scale Validation

Study 1 generated six causes of Chinese employees' complaints in the workplace. However, it was generated from a convenient sample of employees. It was necessary to examine the generalizability of the six-dimensional solution among different samples. Study 2 aims to do this based on a different employee sample by employing confirmatory factor analysis.

### Subjects

A survey comprising the six-dimensional measures of the causes of complaints was conducted among companies in a province of northeastern China. The snowball sampling technique was employed in the data collection. Specifically, ten MBA students from one of the leading universities in northeastern China were selected to help with the questionnaire collection. The selection criteria of these MBA students were (a) being willing to help us collect the questionnaires, (b) working for a company but not a nonprofit organization, and (c) holding a management position in their company. They were first trained for the purposes of the survey and in questionnaire administration techniques, such as not letting the subjects know the purposes of the study and allowing them to complete the questionnaire individually and without interference. Then, 40 printed questionnaires were assigned to each MBA student, and they were asked to return the questionnaires in 3 days. Four hundred questionnaires were distributed, and we received a total of 349 valid responses, for a valid response rate of 87.25%. All of the subjects in this study also provided informed consent. Within the sample of valid responses, 65.9% of respondents were men, 27.5% were women, and 6.6% did not report their sex; 37.8% had a junior college degree, 41.8% had a bachelor's degree, 8.3% had a master's degree or above, and 12% did not report their education level; 28.1% worked in services, 20.3% in manufacturing, 19.2% in education and training, 5.2% in IT, 2.0% in energy, and 25.2% in other industries; 42.1% were ordinary staff, 24.1% worked in a technical position, 17.2% worked in a junior management position, 7.4% worked in an intermediate position, 2.0% worked in a senior position, and 7.2% did not report their positions. Their ages ranged from 20 to 60 (*M*_*age*_ = 35.12, *SD* = 8.35).

### Measures

To reduce subjects' burden in completing the questionnaire measuring the causes of complaints, only the 4 items with high factor loadings were retained for the “dissatisfaction with interpersonal relationships” and “perceived misfit regarding work content” dimensions. Thus, the measurement of the causes of complaints consisted of 22 items: factors 1 to 4 included 4 items each, whereas factors 5 and 6 included 3 items each. Subjects indicated their attitudes toward each item on a 7-point scale, with 1 = *completely disagree* and 7 = *completely agree*.

### Results and Discussion

Referring Zhang et al. (2011), various confirmatory factor analyses were performed based on the LISREL computer program, version 8.7 (Jöreskog and Sörbom, 1996) to compare the proposed model with alternatives to determine the best fitting model. Given that the Pearson correlation coefficients between original factors 1 and 6, 1 and 3, and 3 and 6 in Study 1 were high (i.e., over 0.5), five competing models were tested in these analyses: three five-factor models with the integration of original factors 1 and 6, 1 and 3, 3 and 6; a four-factor model in which original factors 1, 3 and 6 were integrated; and the proposed model with six correlated factors (see Table 3). The maximum likelihood method was used in these analyses. A covariance matrix of the 22 items was generated as input data (cf. Zhang et al., 2011).

**Table 3:** LISREL model comparison for the items on the causes of employee complaints.

**Models**	**χ^2^**	**df**	**χ^2^/df**	**GFI**	**AGFI**	**NFI**	**TLI**	**SRMR**	**RMSEA**
Five factors (factors 1 and 6 were integrated)	830.91	199	4.18	0.82	0.77	0.96	0.97	0.059	0.097
Five factors (factors 1 and 3 were integrated)	950.43	199	4.78	0.80	0.74	0.95	0.96	0.061	0.106
Five factors (factors 3 and 6 were integrated)	1,019.24	199	5.21	0.79	0.73	0.95	0.96	0.061	0.110
Four factors (factors 1, 3 and 6 were integrated)	1,175.31	203	5.79	0.76	0.70	0.95	0.95	0.065	0.119
Six correlated factors	552.18	194	2.84	0.87	0.83	0.97	0.98	0.056	0.074

Seven fit indicators generated by LISREL software, version 8.7, were used to compare the six models. The first is the ratio of Chi-square to its degree of freedom, which tests the extent to which the sample data support the hypothesized model; the value of this ratio between 2 and 3 is deemed acceptable (Tabachnick and Fidell, 2019). The second indicator is the goodness-of-fit index (GFI), which assesses the degree to which the observed covariance matrix fits the hypothesized model. The third indicator is the adjusted goodness-of-fit index (AGFI), which adjusts the GFI according to the number of items of each latent variable. A GFI over 0.9 and an AGFI over 0.8 indicate an acceptable model fit (Tabachnick and Fidell, 2019). The fourth indicator is a normed fit index (NFI), which analyzes the chi-square discrepancy between the hypothesized model and null model; when this index is above 0.9, there is a good fit of the examined model (Tabachnick and Fidell, 2019). The fifth indicator is the Tucker Lewis Index (TLI), which is similar to the NFI. However, this indicator is lower, and the proposed measurement model is regarded as less acceptable. TLI values >0.95 are considered to be a good fit (Hu and Bentler, 1999). The sixth index is the standardized root mean square residual (SRMR) which describes the standardized difference between the observed correlation and the predicted correlation. SRMR values <0.06 are considered a good fit, and those <0.07 are considered acceptable (Hu and Bentler, 1999). The final index is the root mean squared error of approximation (RMSEA), which indicates the discrepancy between the covariance structure set in the model and the covariance structure observed in the sample data. If this index is below 0.08, there is an acceptable fit to the data (Tabachnick and Fidell, 2019).

Compared to the four alternative models, the results shown in Table 3 indicate that the proposed model of six correlated factors best fits the sample data. The χ^2^/df ratio was 2.84, GFI equaled 0.87, AGFI was greater 0.8, NFI equaled 0.97, TLI equaled 0.98, the SRMR equaled 0.056 and the RMSEA equaled 0.074. Only the GFI index was slightly smaller than the required value, and all of the other indices meet the requirements. In the six-correlated-factors model, the 22 items had loadings ranging from 0.70 to 0.90 that loaded on factors 1 through 6.

Additionally, the configural invariance test (Milfont and Fischer, 2010) was performed to determine whether the six-factor solution of the causes of employee complaints was stable across the samples of Study 1 (*N* = 268) and Study 2 (*N* = 349). We conducted this test using multiple-group confirmatory factor analysis with LISREL 8.7 (Milfont and Fischer, 2010). Specifically, we first conducted two confirmatory factor analyses with samples from Study 1 and Study 2. Then, we performed multiple-group confirmatory factor analysis with a combined sample of the two studies. The results of the fit indices of the three models were shown in Table 4. The results showed that the six-factor solution of the causes of employee complaints fit well for the sample from Study 1, the sample from Study 2, and the combined sample from both studies. For all three models, the χ^2^/df ratio was <3, NFI was >0.9, TFI was >0.95, SRMR was ≤ 0.07 (acceptable level, Hu and Bentler, 1999), and RMSEA was <0.08. These indices indicate that the six-factor solution was equal across the two samples (Milfont and Fischer, 2010).

**Table 4:** Fit indices for configural invariance testing toward the six-factor solution.

**Models**	**χ^2^**	**df**	**χ^2^/df**	**GFI**	**NFI**	**TLI**	**SRMR**	**RMSEA**
M_0−Sample of Study 1_	398.93	194	2.06	0.88	0.94	0.96	0.070	0.064
M_0−Sample of Study 2_	552.18	194	2.84	0.87	0.97	0.98	0.056	0.074
M_1−Combined sample of two studies_	951.11	388	2.45	0.88	0.96	0.97	0.069	0.069

## General Discussion

This study focuses on the causes of Chinese employees' complaints in their workplace. Although prior studies based on Western cultures documented many causes of employees' complaints, little research has specifically investigated the causes of Chinese employees' complaints. Based on two Chinese employee samples, this study developed and validated the dimensions of the causes of employee complaints in the workplace based on the theory of work adjustment.

Specifically, we first obtained 49 items about the causes of complaints following the rigorous item generation procedures recommended by the extant literature. Then, an exploratory factor analysis of the causes of employees' complaints in Study 1 generated six components that explained 65.85% of the variance. Using items with high factor loadings in Study 1, we performed confirmatory factor analysis in Study 2, and the results showed that the six correlated factor solutions fit the sample data well. Further configural invariance test verified the equivalence of the six-factor solution across the two samples of Study 1 and Study 2. These results suggested that the causes of Chinese employees' complaints included dissatisfaction with: (a) interpersonal relationships with colleagues; (b) management system; (c) work conditions; (d) authoritarian leadership; and perceived misfit regarding: (e) work content; and (f) job responsibilities. The former four can be classified as P-E interactional factors, and the latter two are P-E misfit factors. Moreover, this study indicates that the first factor, i.e., employees' dissatisfaction with interpersonal relationships with colleagues, contributed the most to the variance in their complaints.

### Theoretical Contributions

This study contributes to the literature in the following ways. First, it identifies four P-E interactional factors and two P-E misfit factors that lead to Chinese employees' complaints in the workplace. Some of these causes—such as dissatisfaction with work conditions, perceived misfit regarding job responsibilities and work content—have been documented in prior studies (e.g., Bacharach and Bamberger, 2004; Furåker, 2009; Branham, 2012; Warr and Inceoglu, 2012). This study reveals that dissatisfaction with interpersonal relationships with colleagues, management systems, and authoritarian leadership are prominent causes in the Chinese context.

In particular, this study reveals that the P-E interactional factor—dissatisfaction with interpersonal relationships with colleagues—is the leading cause (accounting for 34.25% of the variance) of Chinese employees' complaints. In contrast, prior studies based on Western cultures (cf. Piasentin and Chapman, 2006; Westover and Taylor, 2010; Rubin and Edwards, 2020) widely suggested that the misfit between employees' need and organizations' reward was a principal factor in employees' complaints. China is a collectivism-oriented country, and Chinese individuals are highly concerned about their ties both to others and to their organizations (Felfe et al., 2008). Thus, Chinese employees are sensitive to interpersonal relationships with their colleagues (Powell et al., 2009). Once negative feelings related to interpersonal relationships have been experienced (e.g., feeling isolated or neglected by colleagues), Chinese employees will display noticeable reactions such as complaints.

Moreover, the findings indicate that dissatisfaction with the management system is also a China-specific cause of employees' complaints. China remains a developing country at least in terms of business management. There are many imperfections in both state and private-owned companies' management systems. All of the problems in job descriptions, objectives, responsibilities, procedures, etc., make employees feel dissatisfied and potentially likely to complain.

Finally, this study reveals that dissatisfaction with managers' authoritarian leadership is also a China-specific cause of employee complaints. China's recent modernization has reduced its social power distance (Xi et al., 2021), which reflects the extent to which less powerful employees of organizations accept and expect that power is distributed unequally (Wang et al., 2020). This means that contemporary Chinese employees consider that an organization's power should be distributed in a relatively equal manner. In fact, participatory management (encouraging employees to engage in organizational decisions) was recommended by prior studies (e.g., Cheung and Wu, 2014). Nevertheless, many managers in China still adopt an authoritarian leadership style; they are inclined not to listen to subordinates when making decisions (Schuh et al., 2013). This approach will make employees feel dissatisfied and complain.

### Practical Implications

First, our findings have implications for both Chinese and foreign employers who employ Chinese employees regarding the causes of employee complaints. In addition to causes such as dissatisfaction with work conditions, a perceived misfit with job responsibilities and work content that are common among Chinese and Western employees, there were some different causes for Chinese employees' complaints. Dissatisfaction with interpersonal relationships with colleagues was the leading factor for Chinese employees. Thus, in the process of investigating employees' complaints or grievances, managers or counselors should pay special attention to this factor.

Second, this study has specific implications for Chinese employers. Given that dissatisfaction with the management system and authoritarian leadership are China-specific causes of employee complaints, improving management qualities following current management science and avoiding authoritarian leadership can help relieve employee complaints in the workplace.

Finally, this research developed and validated dimensions of complaint causes and measurements among Chinese employees. When suffering negative outcomes such as absenteeism, turnover, lowered productivity, and/or disruptive workplace behaviors (Walker and Hamilton, 2011a) from Chinese employees, employers or their counselors could use this measurement to identify the potential dissatisfaction factors of their Chinese employees.

### Limitations and Future Directions

This study has limitations. First, given that we focused on investigating the causes of complaints among Chinese employees, convergent and discriminant validity with other similar constructs, such as interpersonal conflict at work (Bianchi, 2015) and abusive supervision (Camps et al., 2016), were not examined. Future research is needed to examine whether each factor generated in this study is a unique or overlapping construct in predicting employees' complaints. Second, we discussed the differences between Chinese and Western employees involving the causes of complaints but did not empirically investigate possible differences based on cross-cultural samples. Further studies are also needed to examine the differences in complaint causes between Chinese employees and their counterparts in developed Western countries.

## Conclusion

Workplace complaints are common among Chinese employees and are usually accompanied by negative outcomes at both the individual and collective levels. However, few prior studies have empirically examined the causes of Chinese employees' workplace complaints. Drawing on the theory of work adjustment, the current research sheds light on this issue. Through two studies, our findings reveal six causes of Chinese employees' complaints in the workplace. Four of them are P-E interactional causes, i.e., dissatisfaction with: (a) interpersonal relationships with colleagues; (b) management system; (c) work conditions; and (d) authoritarian leadership; and the other two are P-E misfit factors, i.e., perceived misfit regarding: (e) work content; and (f) job responsibilities. Dissatisfaction with interpersonal relationships with colleagues is the number one cause of workplace complaints among Chinese employees. This research also developed and validated a scale for measuring the causes of workplace complaints among Chinese employees. Our findings and the developed scale could help business managers or consultants to identify the causes of workplace complaints among their Chinese employees and thus help them to address these complaints.

## Data Availability Statement

The raw data supporting the conclusions of this article will be made available by the authors, without undue reservation.

## Ethics Statement

Ethical review and approval was not required for the study on human participants in accordance with the local legislation and institutional requirements. The patients/participants provided their written informed consent to participate in this study.

## Author Contributions

SY was in charge of the paper writing. YG was in charge of data collection and processing. Both authors contributed to the article and approved the submitted version.

## Funding

The authors would like to acknowledge funding from the National Social Science Foundation of China (18BGL133).

## Conflict of Interest

The authors declare that the research was conducted in the absence of any commercial or financial relationships that could be construed as a potential conflict of interest.

## Publisher's Note

All claims expressed in this article are solely those of the authors and do not necessarily represent those of their affiliated organizations, or those of the publisher, the editors and the reviewers. Any product that may be evaluated in this article, or claim that may be made by its manufacturer, is not guaranteed or endorsed by the publisher.

## Footnotes

^1^All of the original dimensional items in Table 1 were generated in Chinese. These items were translated according to the procedure recommended by Brislin (1970). The authors and two other Ph.D. students translated these items into English, and an associate professor who earned his Ph.D. in Australia were asked to back-translate the English version items into Chinese. Discrepancies in the translations were carefully checked and corrected.
